# The impact of patient preference in the treatment algorithm for recurrent/metastatic head and neck squamous cell carcinoma

**DOI:** 10.1007/s11547-022-01509-1

**Published:** 2022-06-25

**Authors:** Viola Salvestrini, Carlotta Becherini, Isacco Desideri, Luisa Caprara, Matteo Mariotti, Marco Banini, Nicola Pierossi, Vieri Scotti, Lorenzo Livi, Pierluigi Bonomo

**Affiliations:** 1 Radiation Oncology, Azienda Ospedaliero-Universitaria Careggi, University of Florence, largo Brambilla 3, 50134 Florence, Italy; 2 Otolaryngology Head & Neck department Meyer Hospital, Florence, Italy

**Keywords:** Immunotherapy, Pembrolizumab, Recurrent or metastatic HNSCC, Patient’s preference, Quality of life

## Abstract

The advent of immune checkpoint inhibitors for recurrent/metastatic head and neck squamous cell carcinoma (RM-HNSCC) has revolutionized the standard of care approach in first-line treatment. The heterogeneity of disease presentation and treatment-related toxicities can be associated with suboptimal patient compliance to oncologic care. Hence, prioritizing quality of life and well-being are crucial aspects to be considered in tailoring the best treatment choice. The aim of our work is to present a short report on the topic of the patient’s preference in regard to treatment and its consequences on quality of life in the recurrent/metastatic setting. According to the literature, there’s an unmet need on how to assess patient attitude in respect to the choice of treatment. In view of the availability of different therapeutic strategies in first-line management of RM-HNSCC, increasing emphasis should be put on integrating patient preferences into the medical decision-making.

## Abstract (it)

L'avvento degli inibitori del checkpoint immunitario per il carcinoma squamocellulare ricorrente/metastatico del distretto testa e collo (RM-HNSCC) ha rivoluzionato l'approccio standard di cura nel trattamento di prima linea di questa malattia. L'eterogeneità di presentazione del RM-HNSCC e le tossicità correlate al trattamento possono essere associate a una compliance non ottimale del paziente alle cure oncologiche. Per tale ragione, qualità della vita e benessere del paziente sono aspetti cruciali da considerare per definire la migliore scelta di trattamento. Lo scopo del nostro lavoro è presentare una panoramica sul tema della preferenza del paziente rispetto al trattamento e delle sue conseguenze sulla qualità della vita in ambito di malattia recidivante/metastatica. Secondo la letteratura infatti, esiste un'esigenza concreta su come valutare l'atteggiamento del paziente rispetto alla scelta del trattamento. In considerazione della disponibilità di diverse strategie terapeutiche nella gestione di prima linea di RM-HNSCC, si dovrebbe dunque porre maggiore enfasi sull'integrazione delle preferenze del paziente nell’individuazione dell’iter terapeutico.

## Introduction

Head and neck squamous cell carcinoma (HNSCC) is the seventh most common cancer worldwide [1]. Distant metastases and/or local recurrence after primary curative treatment occur in about half of patients with locally advanced HNSCC. Approximately 5% of patients have upfront metastases [2]. The prognosis of recurrent/metastatic HNSCC (RM-HNSCC) remains extremely poor with a median overall survival (OS) of about one year [3–5]. Until the publication of the results of Keynote-048 trial, the standard of care of RM-HNSCC was cetuximab plus chemotherapy with platinum and 5-fluorouracil [4]. Immunotherapy for RM-HNSCC has provided promising results and the “one-size-fits-all-approach” in first-line therapy has recently changed [5, 6]. Notwithstanding the progress achieved in the selection of first-line therapy, the treatment for RM-HNSCC remains an open question due to the heterogeneity of patients’ characteristics, symptoms burden and disease presentation. All these factors—performance status, age, comorbidities, need of quick tumor response and PD-L1 Combined Positive Score (CPS)—are simultaneously the main aspects to be considered in the decision-making. However, recurrent and metastatic head and neck patients may suffer from complications such as infections, nutritional issues and voice alterations that negatively affect their quality of life. Moreover, the adverse events of therapy are crucial aspects to be taken into account in the management of HNSCC patients. The aggressiveness of treatment-related toxicities can contribute to refusal of therapeutic options and/or premature interruption of oncologic care, especially in vulnerable populations [7–11]. The aim of our work is to present a critical overview on the topic of the patient’s preference in regard to treatment and its consequences on quality of life in the recurrent/metastatic setting.

### Preferences and priorities of head and neck cancer patients

HNSCC poses a significant burden on HRQoL. Impairments of the anatomic structures involved in breathing, speech and swallowing can occur as the results of the disease itself or can be caused by aggressive treatments [12, 13]. With this regard, patients with HNSCC commonly develop remarkable social isolation and psychological distress [14]. Maintaining HRQoL and psychological well-being are crucial aspects in the management of treatment and independent prognostic factors for survival, especially in RM-HNSCC patients [15–20].

This highlights the relevance of the patients’ perspective as a variable outcome in addition to survival, recurrence or physical impairment [10]. Even though the questionnaire-based evaluation of HRQoL is mostly adopted in clinical trials, we, as others, truly believe that in clinical practice the perception of disease and treatment of each patient remain challenging to investigate. In addition to the negative effects of treatments on HRQoL, patients with RM-HNSCC have to deal with their poor prognosis. Recently, pembrolizumab in combination with platinum/5-FU and pembrolizumab monotherapy have yielded a significant survival benefit compared to the EXTREME regimen in first-line treatment for RM-HNSCC [5, 21]. In addition to the PD-L1 combined positive score (CPS) result, in PS 0–1 patients the choice between the combination of pembrolizumab plus chemotherapy and immunotherapy alone is mostly driven by the need of a rapid tumor shrinkage. Therefore, for RM-HNSCC with persistence of locoregional disease, high risk of airways obstruction and consequent need of quick tumor response the combination of chemotherapy plus immunotherapy can be recommended as preferential option [6].

However, the pembrolizumab monotherapy can be an optimal choice for “hard-to-approach” RM-HNSCC patients thanks to its triweekly schedule, short infusion time for drug administration, no need for central venous access devices and good tolerability profile.

**Fig. 1:**
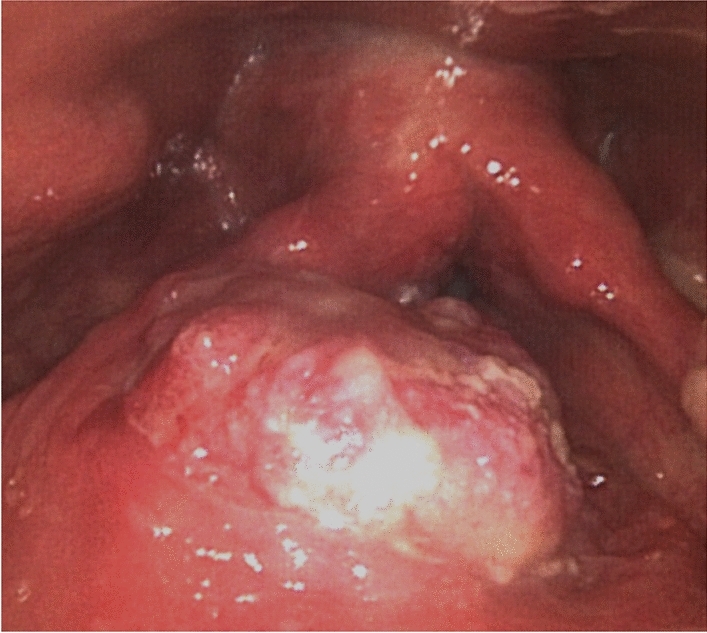
Fiberoptic endoscopy at the first clinical evaluation

**Fig. 2:**
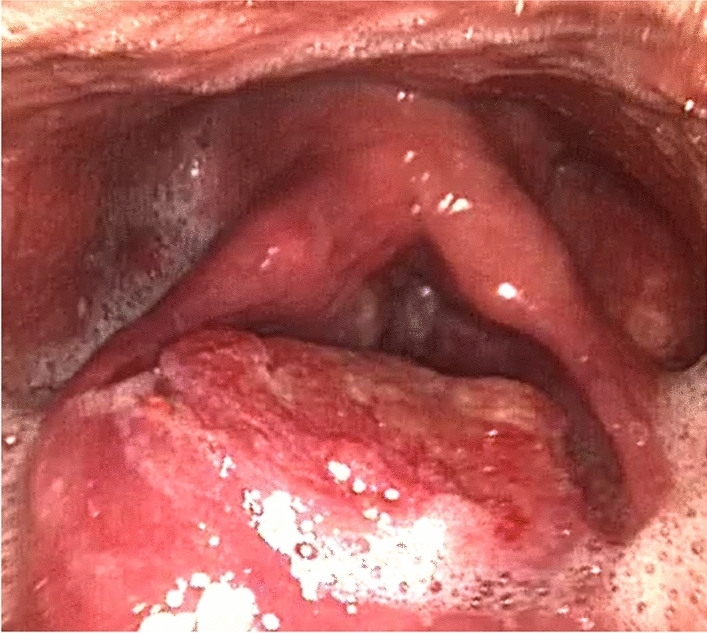
Fiberoptic endoscopy after seven cycles of pembrolizumab

Of note, we recently examined a paradigmatic example of a RM-HNSCC patient treated with pembrolizumab monotherapy due to the refusal of chemo-based treatment options. The patient presented with a SCC of the larynx (Fig. 1). Contrast-enhanced (CE) computed tomography (CT) scan and/or magnetic resonance imaging (MRI) and positron emission tomography (PET) imaging were recommended as imaging workup [22, 23] but the patient refused to perform any further diagnostic-therapeutic approach. Two months after the refusal, he presented with progressive disease (cT4aN2cM1, IVc stage) with CPS of 1 or more preferring a “chemo-free” approach with pembrolizumab monotherapy. Currently, he has received 7 cycles of immunotherapy with unaffected well-being and clinical stability of disease (Fig. 2).

Although disease- and treatment-related aspects represent the key components in the management of RM-HNSCC, patient-related factors such as availability of caregivers, patients’ quality of life, need and preferences should be simultaneously considered [11] and our experience is a representative example of this need.

Increasingly, patients are involved in medical decisions about oncological treatments using tradeoffs between survival benefits and exceeding morbidity from treatment [24, 25]. In HNSCC, the importance of providing increased survival while maximizing patients' quality of life and well-being has been known for many years. More than 40 years ago, McNeil et al. [25] published attitudinal data toward survival and artificial speech of 37 volunteers with stage T3 laryngeal cancer. Up to 20 per cent of the interviewed patients would choose radiation therapy instead of surgery in order to preserve voice suggesting that treatment choice should be made on the basis of preference about the quality as well as the quantity of survival.

Although data about patients' perspectives of treatments remain still limited, it is well known that psychosocial distress caused by HNSCC diagnosis and treatment-related toxicities may lead patients to refusal or interruption of oncological care. In recent decades, some authors focused their effort on analyzing the subset of head and neck patients who are inclined to refuse treatment reporting that 1.3–1.7% of patients with HNSCC refused surgical- and/or radiotherapy-based definitive therapy [15–26].

According to the analysis of 797 patients with unresectable and metastatic tumors who did not perform any sort of treatment in a single center experience, 19% of patients refused therapy based on their personal choice [27]. Moreover, Choi et al. [28] documented that 32.2% did not receive any treatment and identified the advanced age, worse socioeconomic status and lip/oral cavity tumor as the main risk factors related to patient refusal of care.

A retrospective experience of 35,834 patients reported a rate of untreated patients of 10%. The main factors associated with treatment failure were the higher stage of the disease, pharyngeal site and black race [29]. A more recent analysis published by Cheraghlou et al. [30] including 36,261 patients with resectable oral cavity cancer documented a rate of treatment failure of about 1%. Similarly, the higher primary tumor and nodal stage, age of 75 years or older, insurance status and treatment at low/intermediate volume facilities were associated with a patient's likelihood of refusing treatment.

However, there is a large unmet medical need as well as a lack of evidence on tools to assess the preferences of HNSCC patients in regard to treatment and its consequences on several life aspects (Table 1).

**Table 1:** Studies assessing preferences and priorities of head and neck cancer patients

Studies assessing preferences and priorities of head and neck cancer patients							
Study [Ref]	Institution	Date	N° of HNC pts	No of laryngeal cancer pts (%)	RM HNC patients	Instrument	Main findings
Jalukar [34]	University of Iowa, USA	1998	49	NS	NI	TTO	Healthcare professionals and patients have similar attitudes regarding the desirability of potential health-state outcomes within the HNC-specific domains of eating, speech, appearance and breathing
Sharp [35]	University of Chicago, USA	1998	20	2 (10%)	NI	Ranking (CPS, design of the scale)	Being cured/live longer was first priority
List [33]	Multi-institution, USA	2000	131	36 (27%)	NS	Ranking (CPS, FACT-HN, PSSHN)	Cure ranked first for 75% of pts, then living long, having no pain, energy, swallowing, voice and appearance
Gill [36]	Newcastle, UK	2007	30	NS	NI	Ranking (CPS, Ottawa DRS)	Being cured/live longer uniformly ranked first, pain and swallowing items ranked next, but with varying scores
Kanatas [37]	Liverpool, UK	2011	447	186 (42%)	NI	Ranking (PCI, UW-QoL)	Fear of recurrence was the first concern, then issues more specific to each disease such as speech (larynx) and salivation (oropharynx) Variation by age (less fear of recurrence in among elderly pts)
Tschiesner [32]	Munich, Germany	2013	300	130 (43%)	NS	Ranking (ICFHNC)	Survival ranked first (but only by 58% of pts), all expenses for cancer treatment being covered 2nd (51%), being able to continue performing all daily life activities well (50%)
Windon [38]	Baltimore, USA	2020	150	18 (12%)	NS	Ranking (CPS)	Top three priority were cure, survival and swallow. Prioritization of cure, survival and swallow was similar by human papillomavirus (HPV) tumor status. By increasing decade of age, older participants were significantly less likely than younger to prioritize survival
Bonomo [31]	7 institutions worldwide	2020	111	15 (13.5%)	20 (23%)	Ranking (list of issues from a phase I-II study)	Cure of disease, survival-live as long as possible and trusting in health care providers were the 3 most common priorities
Mc Neil [25]	Boston, USA	1981	37	37 (100%)	NI	TTO	20% of pts would choose radiation therapy instead of surgery in order to preserve voice
Otto [39]	UT San Antonio, USA	1997	46	46 (100%)	NI	TTO	Only 20% of pts willing to trade survival for function, by a mean of 5.6 years
Van der Donk [40]	Rotterdam, Netherlands	1995	20	10 (50%)	NI	TTO, SG, RS, DC	Most respondents preferred RT alone; utilities always higher for RT alone than TL

*CPS*, Chicago Priority Scale; *DRS*, Decision Regret Scale; *FACT-HN*, Functional Assessment of Cancer Therapy-Head and Neck; *HNC*, head and neck cancer; recurrent/metastatic HNC (RM HNC); *ICF-HNC*, International Classification of Functioning, Disability and Health Core Set for Head and Neck Cancer; *PSS-HN*, Performance Status Scale for Head and Neck Cancer; *PCI*, patient concerns inventory; *pts*, patients; *S*, subject; *UW-QOL*, University of Washington Head and Neck Cancer Questionnaire; *DC*, direct comparison; *HNC*, head and neck cancer; *RS*, rating scale; *SG*, standard gamble; *TL*, total laryngectomy; *TTO*, Time Trade Off; *RT*, radiotherapy; *NI*, not included; *NS*, not specified

Currently, the methods used to assess the preferences of patients with head and neck cancer are heterogeneous and the gold standard is still missing.

Interestingly, a recent prospective phase I–II study has been published with the purpose to develop a HNSCC patients’ preference questionnaire. Among the final list of items for patients’ preferences, “cure of disease,” “survival-live as long as possible” and “trusting in health care providers” were the three most common priorities in 87.3%, 73.6% and 59.1% of patients, respectively [31].

Similarly, some authors (Table 1) focused their investigation on identifying the checklist of priorities for this population in regard to long-term treatment effects. Although the item of “being cured/living longer” was uniformly ranked first [32–35], in the Gill et al. [36] assessment also no pain and swallow preservation were indicated as the main priorities for HNSCC interviewed patients. Additionally, Kanatas et al. [37] showed that also fear of recurrence was common to all clinical groups and speech issues were much more considered by laryngeal cancer patients than other subgroups of HNSCC. In a more recent publication, Windon et al. [38] investigated the perspective and preference of a prospective cohort of 150 HNSCC patients, confirming that oncological treatments and benefits in terms of survival were the two most priorities.

Although we found a wide heterogeneity of data from the head and neck cancer patients, remarkable findings were reported in the papers of larynx preservation. This subgroup of site-specific studies [25, 39, 40] interviewed the patients using similar questions in regard to the utility of laryngectomy health state or the tradeoff between survival and laryngectomy. Moreover, the patients’ preference tool used by the authors was mostly time trade-off and the results were much more homogeneous. Contrary to the previous papers assessing ranking of preference and priority, the main findings from studies on laryngeal preservation suggested that ‘‘live longer” is not the main expectancy. Laryngeal cancer patients mostly preferred radiation treatment alone or in combination with chemotherapy than laryngectomy in order to preserve voice and speech [25, 39–42]. Notably, the inclusion of RM HNSCC in the aforementioned experiences is underrepresented (22% of patients in the subgroup of palliative treatment [31]) or completely missing.

## Conclusions

Ideally, the development of a comprehensive questionnaire assessing the heterogeneous domains of preference may allow to fully integrate also the RM-HNSCC patients’ priorities in the medical decision. Prospective studies designed to integrate patients’ needs and preferences in the optimal choice of first-line treatment are warranted.

## Data Availability

Data that support the findings of this study are available from the corresponding author upon reasonable request.
